# Land-atmosphere feedbacks drive dryland drought and expansion under climate warming

**DOI:** 10.1016/j.xinn.2025.100863

**Published:** 2025-02-26

**Authors:** Lei Gu, Dominik L. Schumacher, Hui-Min Wang, Jiabo Yin, Erich M. Fischer

**Affiliations:** 1 Institute for Atmospheric and Climate Science, ETH Zurich, 8092 Zurich, Switzerland; 2 Department of Civil and Environmental Engineering, National University of Singapore, Singapore 117576, Singapore; 3 State Key Laboratory of Water Resources Engineering and Management, Wuhan University, Wuhan, Hubei 430072, P.R. China

## Main text

Drylands cover ∼45% of Earth’s land surface, support ∼40% of the global population, and harbor ∼30% of endangered species. However, anthropogenic climate change increasingly dries drylands through multiple processes and feedback mechanisms. From a thermodynamic perspective, global warming elevates atmospheric vapor pressure deficit (VPD) in drylands, accelerating moisture loss from vegetation and bare soils, thereby exacerbating aridity in drylands. This mechanism is further amplified by local land-atmosphere feedbacks: soil desiccation and vegetation dry out typically result in a reduced evaporation fraction. This, in turn, decreases the relative humidity yet further boosts the water demand of air. Furthermore, human-induced shifts in large-scale atmospheric circulations, reduce local precipitation and further aggravate aridity in subtropical drylands.

Despite various physical processes of dryland changes being documented in recent decades, the role of land-atmosphere feedbacks in shaping drylands’ water supply has remained elusive. In *Science*, Koppa and colleagues^1^ introduced an observationally constrained physical Lagrangian transport model to elucidate how land-atmosphere feedbacks drive the humid-dry transition on our warming Earth. They highlight the crucial role of upwind land-atmosphere feedbacks in regulating downwind hydroclimatic conditions under anthropogenic change over multi-decadal timescales. This influence is also significant for individual events in drylands that are amplified by upwind land-atmosphere conditions. Schumacher et al.^2^ systematically quantified the contribution of land-atmosphere feedbacks in influencing 40 major droughts worldwide, revealing their key role in propagating soil droughts across drylands. Together, we underscore the growing importance of land-atmosphere feedbacks not only in local drylands but also in their broader impacts on downwind regions across different timescales in the context of anthropogenic warming.

## Long-term land-atmosphere feedback enables dryland self-expansion

Numerous studies have shown that climate warming since the 1950s has caused significant drying trends (aridification) and widespread expansion of global drylands, particularly in southern Africa and the Sahel. While dryland expansion and aridification are often attributed to rising VPD in a warming climate—reflecting both the increased evaporation potential and the inability of evaporation to offset atmospheric drying—the detailed mechanisms and quantitative contributions of these drivers remain uncertain.

A universal atmospheric transport model provides an opportunity to unravel the contributions of upwind land-atmosphere feedbacks to dryland self-expansion. This Lagrangian modeling framework traces the trajectories of global air parcels to examine heat and moisture source-receptor relationships. Koppa et al.^1^ applied this approach to identify the influence of land-atmosphere feedbacks on global drylands from 1980 to 2018. In doing so, they demonstrated that land-atmosphere feedbacks—the processes whereby upwind drylands cause aridification in downwind humid regions, termed dryland self-expansion—account for more than half of aridity increases in ∼40% of global humid-dry transition areas.

**Figure 1:**
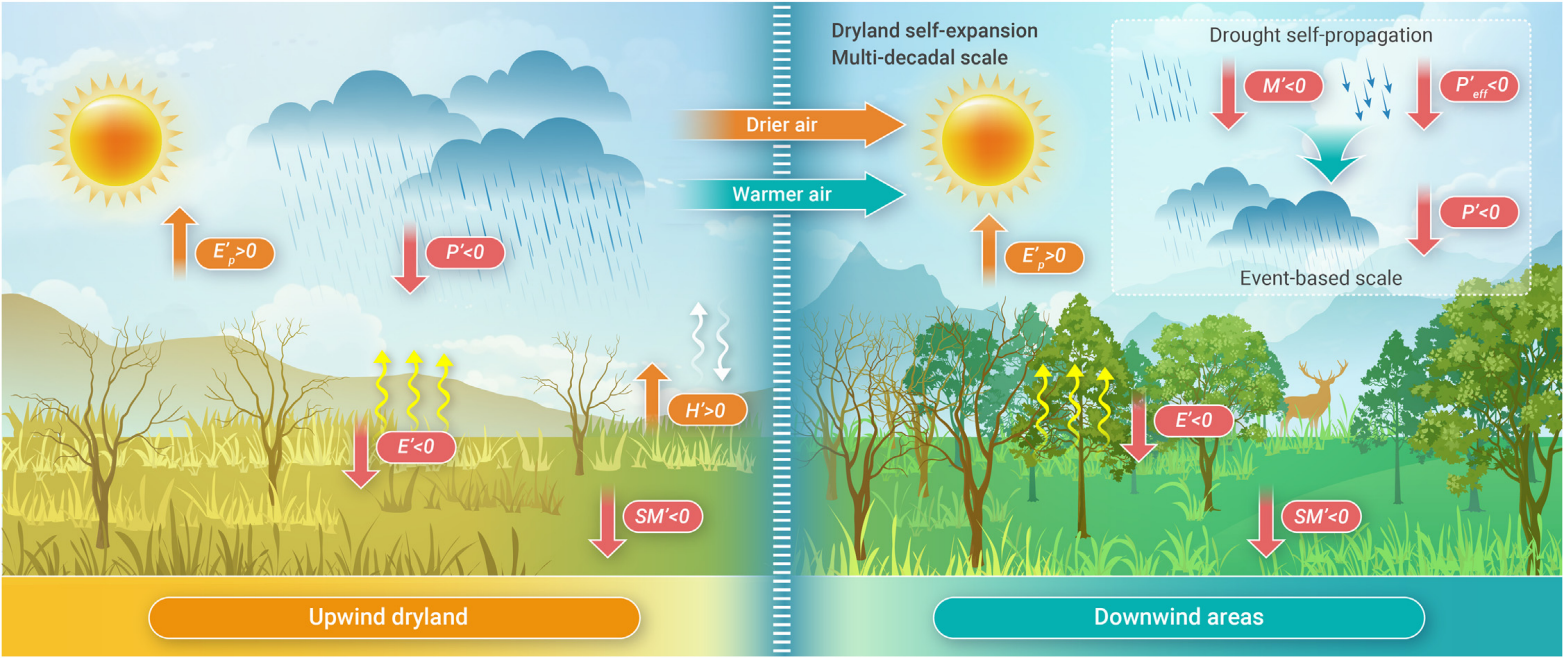
Conceptual representation of land-atmosphere feedbacks drive dryland drought and expansion under climate warming Dryland self-expansion enabled by long-term land-atmosphere feedbacks and drought self-propagation driven by short-term feedbacks. These feedbacks are associated with increases in potential evaporation (*E′*_*p*_ > 0) and reductions in precipitation (*P′* < 0) and soil moisture (*SM′* < 0), which in turn decrease local evaporation (*E′* > 0) and fuel surface sensible heat (*H′* > 0). This causes drier and warmer air traveling to downwind areas. Over the long term, these advection processes result in *P* decreases (*P′* < 0) and *E*_*p*_ surpluses (*E′*_*p*_ > 0) in downwind areas, further reducing downwind *SM* (*SM′* < 0) and *E* (*E′* > 0), contributing to dryland self-expansion. In the short term, the horizontal advection of drier air masses limits water vapor (*M′* < 0) and decreases precipitation efficiency (*P′*_*eff*_ < 0) in downwind regions, thereby decreasing downwind *SM* (*SM′* < 0) and *E* (*E′* > 0) and facilitating drought self-propagation.

In dryland self-expansion, it is the drying of upwind drylands that determines the downwind humid-dry transition, despite the upwind drylands not being the primary moisture supply of downwind areas. Even in the regions where moisture and heat primarily originate from humid areas, such as Australia and eastern Eurasia, upwind dryland drying still drives downwind aridification. The interconnection of moisture and heat transport between drylands and adjacent humid regions fuels these land-atmosphere feedbacks. More explicitly, the drying of drylands limits local evaporation and elevates sensible heating over the long term. This process inflicts downwind aridification through air advection over the course of years and decades, further reducing downwind evaporation and increasing downwind potential evaporation (conceptually linked to enhanced sensible heating), resulting in persistent dryland self-expansion (Figure 1). It should be noted that this upwind dryland aridification includes two pathways—dampened precipitation and rising potential evaporation—and differs across regions. Reduced precipitation in upwind regions is the primary driver of dryland self-expansion in the subtropics and the Southern Hemisphere, while enhanced potential evaporation in upwind areas predominantly drives self-expansion in the Northern Hemisphere mid-latitudes.

As exemplified by their results, climate change promotes dryland aridification and humid-dry transitions through upwind land-atmosphere feedbacks. Additional human-induced warming further fuels the global expansion of drylands by transporting drier and warmer air masses to downwind regions. Land-atmosphere feedbacks in existing drylands thus have a profound role in expanding global drylands, yet this effect is cumulative and only manifests over a sufficiently long time period. This raises the question of whether these upwind land-atmosphere interactions could also impact downwind hydroclimatic conditions in the short term, leading to the spatial expansion of an individual drought.

## Short-term land-atmosphere feedback causes dryland drought self-propagation

To address this question, Schumacher and colleagues employed the Lagrangian atmospheric tracking model to quantify the impact of land-atmosphere feedbacks on an event scale.^3^ Their study revealed that upwind soil droughts in drylands can propagate to downwind areas through land-atmosphere feedbacks, a process termed drought self-propagation. Among the 40 largest drought events in the past four decades, soil droughts in drylands have reduced downwind precipitation by approximately 16% for entire events, with a (monthly) peak reduction of up to 32%.

Traditional drought propagation literature often emphasizes dynamic contributions, such as anomalous atmospheric circulations or variations in regional monsoon strength and movement patterns. These dynamic processes influence moisture and heat advection, alter the conversion of water vapor into precipitation, and ultimately govern drought propagation. Since severe agro-ecological drought is typically initiated by precipitation deficits associated with dynamic systems, the role of upwind land-atmosphere feedbacks in drought propagation has been ignored. Schumacher et al.^2^ provided robust evidence of drought self-propagation via these short-term land-atmosphere feedbacks, with the most susceptible regions consisting of drylands.

When upwind soil droughts occur, evaporation becomes water limited, reducing the moisture supply to downwind areas. But does a decline in upwind moisture always imply downwind moisture deficits and subsequent precipitation shortages? The answer strongly depends on the region of interest and the associated primary source of water vapor. Only in regions with a high regional-scale moisture recycling ratio can upwind soil desiccation lead to downwind precipitation deficits, driving drought self-propagation. For example, in the drylands of southern Africa, diminished moisture recycling during rainfall shortages is known to exacerbate drought conditions, manifesting in drought self-propagation. These effects are far less pronounced in coastal regions with a strong influx of water vapor evaporating from the ocean. This distinction underscores the differences between event-based land-atmosphere feedbacks and multi-decadal feedbacks. Together with the sensitivity of evaporation to water limitation, the dependency of a region on its own supply of precipitation and from neighboring drylands thus governs the contribution of upwind land-atmosphere feedbacks to drought self-propagation.

Whereas long-term remote land-atmosphere interactions drive dryland self-expansion both through enhancing downwind potential evaporation or suppressing precipitation, short-term (event-based) feedbacks primarily enable drought self-propagation through dampening downwind precipitation (Figure 1). This is because individual droughts are dominated by rainfall shortages, which originated from anomalous dynamic systems, and are not directly linked to anthropogenic warming. Consequently, land-atmosphere feedbacks mainly contribute to drought propagation by amplifying the dynamically induced precipitation shortages—reducing water vapor availability and/or precipitation efficiency. However, it is helpful to additionally consider the impacts of heat advection to understand dryland self-expansion due to the fact that human-induced warming strongly intensifies the long-term evolution of potential evaporation.

## Summary

Koppa et al.^1^ suggest that global warming drives the aridification of drylands, initiating dryland self-expansion through long-term land-atmosphere feedbacks. At the event scale, while land-atmosphere feedbacks in upwind regions enable drought self-propagation, global warming amplifies these effects, intensifying drought severity and expanding drought-affected areas. Furthermore, land-use and land-cover changes also modulate the impacts of these feedbacks. For instance, vegetation greening in upwind drylands reduces the surface albedo, which in turn increases sensible heating over the long term and exacerbates downwind humid-dry transition through upwind land-atmosphere feedbacks.^1^ On the other hand, vegetation greening invoked by human activities for parts of (semi-)arid China has been shown to mitigate downwind drought through land-atmosphere feedbacks.^3^ We therefore argue that the downwind effects of vegetation greening through these feedbacks may depend on vegetation types and the temporal scale of feedbacks. Bridging these gaps is crucial for advancing our understanding of how land-atmosphere feedbacks will evolve under the combined influence of global warming and land-use changes.

Even though land-atmosphere feedbacks exert the strongest impact on (semi-)arid environments by exacerbating soil desiccation and thereby promoting both drought self-propagation and dryland self-expansion, such interactions can also influence mid-latitudinal and climatologically more humid regions, contributing to record-breaking heatwaves through boosted heat advection. Notably, upwind land-atmosphere feedbacks considerably exacerbated the 2003 Western European and 2010 Russian mega-heatwaves, accounting for ∼30% of the total advected heat.^4^

The Lagrangian modeling approach offers a robust framework for examining the far-reaching impacts of both long-term and short-term land-atmosphere feedbacks. However, current research focuses on the thermodynamic aspects of these feedbacks, leaving open questions about the potential initiation or correlation between these feedbacks and atmospheric circulation patterns. With rapid advancements in AI-driven deep generative models,^5^ future research could integrate these AI models with the process-based Lagrangian framework to comprehensively explore these feedbacks and interactions between large-scale dynamic systems and thermodynamic changes.

## Acknowledgments

This work was supported by the 10.13039/501100001809National Natural Science Foundation of China (grant no. 52209020) and the 10.13039/100031576Swiss Data Science Center COPE project (grant no. C22-02). J.Y. acknowledges the 10.13039/501100001809National Natural Science Foundation of China (grant nos. 52441902 and W2421111).

## Declaration of interests

The authors declare no competing interests.
